# Pharmacokinetic drug interaction and safety after coadministration of clarithromycin, amoxicillin, and ilaprazole: a randomised, open-label, one-way crossover, two parallel sequences study

**DOI:** 10.1007/s00228-018-2489-2

**Published:** 2018-05-30

**Authors:** Byung Hak Jin, Byung Won Yoo, Jungsin Park, Jung Hye Kim, Jun Yeon Lee, Jae Soo Shin, Min Soo Park

**Affiliations:** 1 Department of Pharmaceutical Medicine and Regulatory Sciences, Colleges of Medicine and Pharmacy, Yonsei University, Incheon, South Korea; 2 Department of Clinical Pharmacology, Severance Hospital, Yonsei University College of Medicine, 50-1 Yonsei-ro, Seodaemun-gu, Seoul, 03722 South Korea; 3 IL-YANG PHARM. Co., Ltd, Yong-in, South Korea; 4 Department of Pediatrics, Yonsei University College of Medicine, Seoul, South Korea

**Keywords:** Proton pump inhibitor, Ilaprazole, Clarithromycin, Amoxicillin, Pharmacokinetics, Drug interaction

## Abstract

**Purpose:**

Ilaprazole, the latest proton pump inhibitor, can be used with clarithromycin and amoxicillin as a triple therapy regimen for eradicating *Helicobacter pylori*. The aim of this study was to evaluate pharmacokinetic drug interactions and safety profiles after coadministration of clarithromycin, amoxicillin, and ilaprazole.

**Methods:**

A randomised, open-label, one-way crossover, two parallel sequences study was conducted in 32 healthy subjects. In part 1, the subjects received a single dose of ilaprazole 10 mg in period 1 and clarithromycin 500 mg and amoxicillin 1000 mg twice daily for 6 days in period 2. In part 2, the subjects received clarithromycin 500 mg and amoxicillin 1000 mg once in period 1 and ilaprazole 10 mg twice daily for 6 days in period 2. In both sequences, the three drugs were coadministrated once on day 5 in period 2. Pharmacokinetic evaluations of ilaprazole (part 1), and clarithromycin and amoxicillin (part 2) were conducted.

**Results:**

Twenty-eight subjects completed the study. For ilaprazole, the peak concentration (C_max_) slightly decreased from 479 (ilaprazole alone) to 446 ng/mL (triple therapy) [Geometric least square mean ratio (90% confidence interval), 0.93 (0.70–1.22)]. The area under the concentration-time curve from 0 h to the last measurable concentration (AUC_last_) slightly increased from 3301 to 3538 μg·h/mL [1.07 (0.85–1.35)]. For clarithromycin, the C_max_ slightly decreased from 1.87 to 1.72 μg/mL [0.90 (0.70–1.15)], and AUC_last_ slightly increased from 14.6 to 16.5 μg·h/mL [1.09 (0.87–1.37)]. For amoxicillin, the C_max_ slightly decreased from 9.37 to 8.14 μg/mL [0.86 (0.74–1.01)], and AUC_last_ slightly decreased from 27.9 to 26.7 μg·h/mL [0.98 (0.83–1.16)]. These changes in the PK parameters of each drug were not statistically significant.

**Conclusions:**

The coadministration of ilaprazole, clarithromycin, and amoxicillin was tolerable and did not cause a significant PK drug interaction. Thus, a triple therapy regimen comprising ilaprazole, clarithromycin, and amoxicillin may be an option for the eradication of *H. pylori*.

**Clinicaltrials.gov** **number**: NCT02998437.

**Electronic supplementary material:**

The online version of this article (10.1007/s00228-018-2489-2) contains supplementary material, which is available to authorized users.

## Introduction

Peptic ulcer disease (PUD), the most common disease related to gastric acid, develops due to an imbalance of aggressive gastric luminal factors (e.g. acid and pepsin) and defensive mucosal barrier function. PUD includes both gastric and duodenal ulcers characterised by epigastric pain, fullness, bloating, early satiety, and nausea [1]. *Helicobacter pylori* infection is found in 60–90% of patients with peptic ulcer [2]. A strong association between *H. pylori* infection and PUD has been shown in epidemiological studies [3, 4]. The eradication of *H. pylori* was found essential for the permanent cure for peptic ulcers [3, 4]. Thus, the eradication of *H. pylori* has become an important factor in PUD treatment.

In addition to PUD, *H. pylori* is associated with many gastrointestinal diseases, such as chronic gastritis, gastric adenocarcinoma, and lymphoma. With the recognition of the clinical importance of *H. pylori*, numerous therapeutic regimens have been evaluated for eradication of *H. pylori*. Although eradication failure due to antibiotic resistance has been a concern recently, clinical guidelines recommend a clarithromycin-containing standard triple regimen as the first-line treatment for *H. pylori* [1, 2]. The standard triple therapy consists of proton pump inhibitors (PPIs), clarithromycin, and amoxicillin [5], which has achieved *H. pylori* eradication rates of 71–80% [6, 7]. However, varying degrees of drug interactions have been observed between coadministered PPIs and antibiotics.

Clarithromycin is a potent cytochrome P450 (CYP) 3A4 inhibitor, and affects most PPIs (such as omeprazole, lansoprazole, and esomeprazole) that are metabolised by CYP3A4 [8–10]. PPIs may, in turn, alter the metabolism of concomitantly administered antibiotics via either CYP enzyme inhibition or a change in the pH-dependent solubility of the drugs [11]. It has been demonstrated that omeprazole attenuates the breakdown of acid-labile antibacterials such as amoxicillin and increases their intragastric concentrations [12]. Based on this finding, it has been speculated that PPIs may increase the bioavailability of acid-labile antibiotics.

Ilaprazole, the latest PPI developed by IL-YANG Pharmaceutical Co., Ltd. (Seoul, South Korea) and approved for the treatment of duodenal ulcers, gastric ulcers, and erosive esophagitis, has the longest half-life among PPIs [13]. Furthermore, it shows greater and prolonged suppression of gastric acid secretion even at a dose of 10 mg that is higher than that achieved with a 20-mg dose of omeprazole [14, 15]. Considering that a double dose of PPIs is superior to a single dose when used as part of triple therapy for *H. pylori* eradication [16], triple therapy including ilaprazole, a more potent PPI, is expected to be effective in *H. pylori* eradication.

In the early period of drug development, in vitro studies reported that ilaprazole was reported from in vitro studies to be mainly metabolised by CYP3A4 among the CYP isoforms, and converted to the major metabolite, ilaprazole sulfone [17–19]. CYP2C19 has been revealed to be rarely involved in ilaprazole metabolism unlike that observed for other PPIs such as omeprazole, esomeprazole, pantoprazole, and lansoprazole [13, 19]. However, a new metabolite, ilaprazole thiol ether, formed via a non-enzymatic metabolic pathway was identified in human studies after ilaprazole administration, and even ilaprazole thiol ether was detected in large or similar amounts compared to those of ilaprazole sulfone [20, 21]. These results indicate that ilaprazole has various metabolic pathways consisting of both CYP-mediated enzymatic and non-enzymatic metabolic pathways, and the non-enzymatic pathway plays a significant role in ilaprazole metabolism. The differences in the metabolic pathway between ilaprazole and other PPIs could also cause differences in drug interactions with coadministered antibiotics for *H. pylori* eradication.

There has been a great demand from clinicians to use ilaprazole in *H. pylori* eradication therapy, but the drug interactions between ilaprazole and coadministered antibiotics in the standard triple therapy (e.g. clarithromycin and amoxicillin) have not yet been clearly determined. In this study we evaluated the pharmacokinetic (PK) drug interaction between clarithromycin, amoxicillin, and ilaprazole intended for a triple therapy regimen for eradication of *H. pylori*.

## Subjects and methods

### Study subjects

Healthy Korean male volunteers who were aged between 19 and 50 years with a body mass index between 18.5 and 25.0 kg/m^2^ were enrolled in the study. Volunteers went through the following screening procedures: taking the medical history, physical examination, 12-lead electrocardiography (ECG), and assessment of clinical laboratory parameters including haematology, blood chemistry, and urine drug screening after they voluntarily signed the informed consent form. The participants were not allowed to consume alcohol, caffeinated beverage, and grapefruit products or smoke during the study period. Any ethical drugs or herbal medicines were prohibited for 2 weeks before the study drug administration.

### Study design, treatments, and administration

This was a randomised, open-label, one-way crossover, two parallel sequences study. A total of 32 subjects were randomised and assigned to one of the two parts. Subjects assigned to part 1 received a single dose of ilaprazole (Noltec Tab., IL-YANG Pharmaceutical Co.) 10 mg in the first period. Then, after a washout period of 10 days, clarithromycin (Klaricid Film Coated Tab., Abbott Korea) 500 mg and amoxicillin (Amoxicillin Cap. 500 mg Chongkundang, Chong Kun Dang Pharmaceutical Co., Korea) 1000 mg were administered twice daily for 6 days in the second period. Ilaprazole 10 mg was coadministered with clarithromycin and amoxicillin on the day 5 of the second period.

**Fig. 1:**
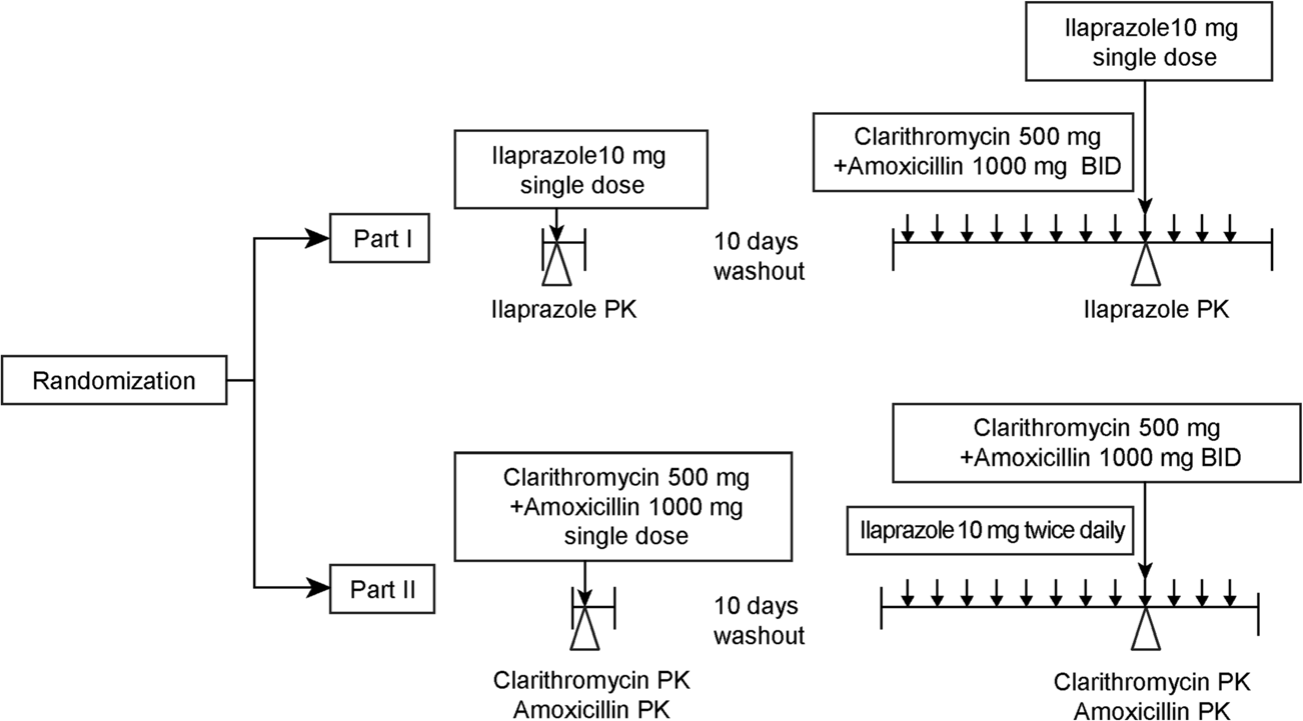
Study design. Notes: Subjects who were assigned to part 1 received ilaprazole 10 mg on day 1 of period 1 and clarithromycin 500 mg and amoxicillin 1000 mg from day 1 to day 6 of period 2 and ilaprazole 10 mg was concomitantly administered on day 5 of period 2. Subjects who were assigned to part 2 received both clarithromycin 500 mg and amoxicillin 1000 mg on day 1 of period 1 and ilaprazole 10 mg from day 1 to day 6 of period 2 and clarithromycin 500 mg and amoxicillin 1000 mg was concomitantly administered on day 5 of period 2

Subjects assigned to part 2 received single doses of both clarithromycin 500 mg and amoxicillin 1000 mg in the first period; after a washout period of 10 days, ilaprazole 10 mg was administered twice daily for 6 days in the second period. Clarithromycin 500 mg and amoxicillin 1000 mg were coadministered with ilaprazole on the day 5 of the second period (Fig. 1). All study drugs were administered via the oral route, and the dosage of each drug was determined based on the results of a previous ilaprazole phase 3 study, treatment guidelines for *H. pylori* eradication, and labels of clarithromycin and amoxicillin [22–27].

The study was approved by the Institutional Review Board of Severance Hospital, Yonsei University College of Medicine, Seoul, Korea, and conducted in accordance with the principles of the Declaration of Helsinki and Good Clinical Practice.

### Blood sample collection and analysis

Serial blood samples were collected in anti-coagulated EDTA-K2 tubes at 0 (pre-dose), 1, 2, 2.5, 3, 3.5, 4, 4.5, 5, 6, 8, 12, 24, and 48 h after dosing of ilaprazole in part 1. The PK sampling points in part 2 were 0 (pre-dose), 0.5, 0.75, 1, 1.5, 2, 2.5, 3, 4, 6, 8, 12, 24, and 48 h after dosing of clarithromycin and amoxicillin. Blood sampling points were determined considering the time to reach peak concentration (T_max_) and elimination half-life (t_1/2_) of ilaprazole, clarithromycin, and amoxicillin [9, 10, 20]. Plasma concentrations of ilaprazole, clarithromycin, and amoxicillin were analysed using liquid chromatography-tandem mass spectrometry (LC-MS/MS). Samples were centrifuged for 10 min under 4 °C at 2000×*g* and stored below − 70 °C until analysis.

For the analysis of ilaprazole, 100 μL of plasma was added to polypropylene tubes and mixed with 10 μL of the internal standard (omeprazole) solution and 1 mL of acetonitrile. The mixture was vortexed for 1 min and centrifuged for 5 min at 11,337×*g*. The supernatant (100 μL) was collected and reconstituted with 600 μL of mobile phase. The supernatant (2 μL) was then injected into an LC-MS/MS system. An LC system (Shiseido Nanospace SI-2, Shiseido, Tokyo, Japan) was used with a mobile phase (10 mM ammonium formate/acetonitrile = 50/50 [*v*/*v*]) at 40 °C. Chromatographic separation was performed at a flow rate of 0.25 mL/min. The transition of ilaprazole was detected at *m*/*z* 367.0 to 184.2 by using an MS/MS system (API4000®, AB SCIEX, Washington DC, USA) in the multiple reaction monitoring (MRM) mode with a positive electrospray ionisation (ESI) source. The calibration curve was linear over the range of 5–1000 ng/mL, and the correlation coefficient (r) was > 0.995.

For the analysis of clarithromycin, 100 μL of plasma was added to polypropylene tubes and mixed with 10 μL of the internal standard (clarithromycin-N-methyl-^13^C, d_3_) solution and 500 μL of acetonitrile. The mixture was vortexed for 1 min and centrifuged for 5 min at 11,337×*g*. The supernatant (50 μL) was collected and reconstituted with 1000 μL of 50% acetonitrile. The supernatant (3 μL) was then injected into the LC-MS/MS system. The LC system was used with a mobile phase (10 mM ammonium formate/acetonitrile = 20/80 [*v*/*v*]) at 40 °C. Chromatographic separation was performed at a flow rate of 0.2 mL/min. The transition of clarithromycin was detected at *m*/*z* 748.4 to 590.4 using an MS/MS system (4000QTRAP, AB SCIEX, Washington DC, USA) in the MRM mode with a positive ESI source. The calibration curve was linear over the range of 10–7000 ng/mL, and the correlation coefficient (r) was > 0.995.

For the analysis of amoxicillin, 100 μL of plasma was added to polypropylene tubes and mixed with 10 μL of internal standard (amoxicillin-d_4_) solution and 500 μL of acetonitrile. The mixture was vortexed for 1 min and centrifuged for 5 min at 11,337×*g*. The supernatant (100 μL) was collected and reconstituted with 100 μL of 50% acetonitrile. The supernatant (5 μL) was injected into the LC-MS/MS system. The LC system was used with a mobile phase (deionised water/acetonitrile/formic acid = 70:30:0.1 [*v*/*v*/*v*]) at 40 °C. Chromatographic separation was performed at a flow rate of 0.2 mL/min. The transition of amoxicillin was detected at *m*/*z* 366.2 to 208.2 using an MS/MS system (4000QTRAP, AB SCIEX, Washington DC, USA) in the MRM mode with a positive ESI source. The calibration curve was linear over the range of 0.1–50 μg/mL, and the correlation coefficient (r) was > 0.995.

The coefficient of variation (CV) represents the overall precision of the assay of the three study drugs, which was < 20.0% for the lower limit of quantification (LLOQ) and 15.0% for other upper concentrations for calibration.

### PK assessment

PK parameters were evaluated using a non-compartmental model of Phoenix WinNonlin® software (version 6.4; Certara, St. Louis, MO, USA). The peak concentration (C_max_) and T_max_ were determined directly from the observed values. The area under the curve from the time of dosing to the last measurable concentration (AUC_last_) was calculated using the linear trapezoidal rule. The terminal elimination rate constant (λ_z_) was estimated by the linear regression analysis of the terminal portion of the log-transformed plasma concentration-to-time profile. The elimination half-life (t_1/2_), apparent plasma clearance (CL/F), and apparent volume of distribution (Vd/F) were calculated using the following equations t_1/2_ = ln(2)/λ_z_, CL/F = dose/AUC_inf_, and Vd/F = dose/(λ_z_ · AUC_inf_), respectively. The AUC of the last dosing time extrapolated to infinity (AUC_inf_) was calculated using the formula AUC_last_ + C_last_/λ_z_, where C_last_ represents the last measurable concentration. The PK drug interactions after triple therapy were evaluated by comparing the plasma concentration-time profiles and PK parameters of each drug.

### Safety assessment

Safety and tolerability were evaluated throughout the study. The assessments included monitoring of all adverse events (AEs) based on physical examinations, vital signs, 12-lead ECG, routine haematology, serum chemistry, and urinalysis. The subjects were instructed to notify the study physicians or nurses of any AEs that occurred during the study. All AEs reported by subjects or detected in the assessments were recorded, and the investigators determined their relationship to the treatment. The severity of the AEs was evaluated based on the Common Terminology Criteria for Adverse Events (CTCAE, version 4.03).

### Statistical analysis

Because this study was explorative and sought to evaluate the PK interactions of the study drugs, it was deemed preferable to use minimum number of subjects. Considering that the power of the parallel study design would be insufficient compared with that of the crossover design, the minimum number of subjects was estimated to be 28, consisting of 14 subjects/per part, and the total number of subjects was 32 (16 subjects/part) under the assumption of a potential drop-out rate of 15%. Descriptive statistics were calculated for baseline demographics, PK parameters, and safety data by part. Baseline demographics of the treatment sequences were evaluated using the Student’s *t* test using the SAS statistical software version 9.4 (SAS Institute Inc. Cary, NC, USA). To compare the PK parameters (C_max_, AUC_last_, and AUC_inf_) between treatments, a general linear mixed effects model was constructed using log-transformed data with treatment as the fixed effect and variance within subjects as a random effect. The geometric least squares mean ratio (GMR) and the 90% confidence interval (CI) of the C_max_, AUC_last_, and AUC_inf_ between the two different treatments of each part were determined. All statistical analyses were conducted using SAS® version 9.4. All statistical tests were two-sided, and a *P* < 0.05 was considered statistically significant.

## Results

### Baseline demographics

A total of 34 subjects were enrolled in this study. Two subjects were dropped before the investigational products were administered because of clinical laboratory test results, and 32 subjects received the investigational products. Each part consisted of 16 subjects, and two subjects were dropped during period 1 because they matched an exclusion criterion (smoking during the PK assessment period) and one was dropped after period 1 owing to herpes zoster in part 1 (in all, three subjects were dropped). One subject was dropped during period 1 because of smoking in part 2. Thus, 28 subjects completed the study and were included in the PK analysis. In contrast, 32 subjects who were administered at least one dose of any investigational product were included in the safety analysis. The demographic information of the study subjects is presented in Supplementary Table 1.

### Pharmacokinetics

**Fig. 2:**
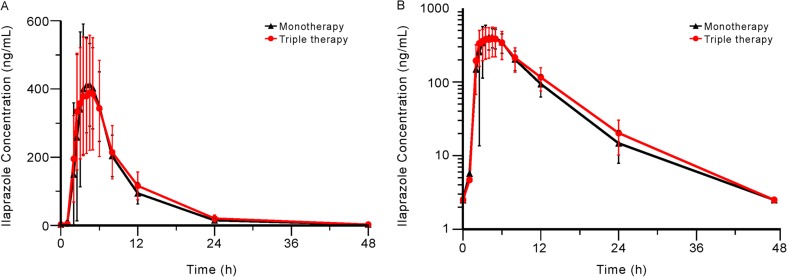
Mean (SD) plasma concentration-time profiles of ilaprazole when administered as monotherapy and as part of triple therapy. **a** Linear scale; **b** semi-logarithmic scale. Abbreviations: monotherapy, administration of ilaprazole 10 mg in period 1 of treatment part 1; triple therapy, administration of ilaprazole 10 mg, clarithromycin 500 mg, and amoxicillin 1000 mg in period 2 of both treatment parts 1 and 2

**Fig. 3:**
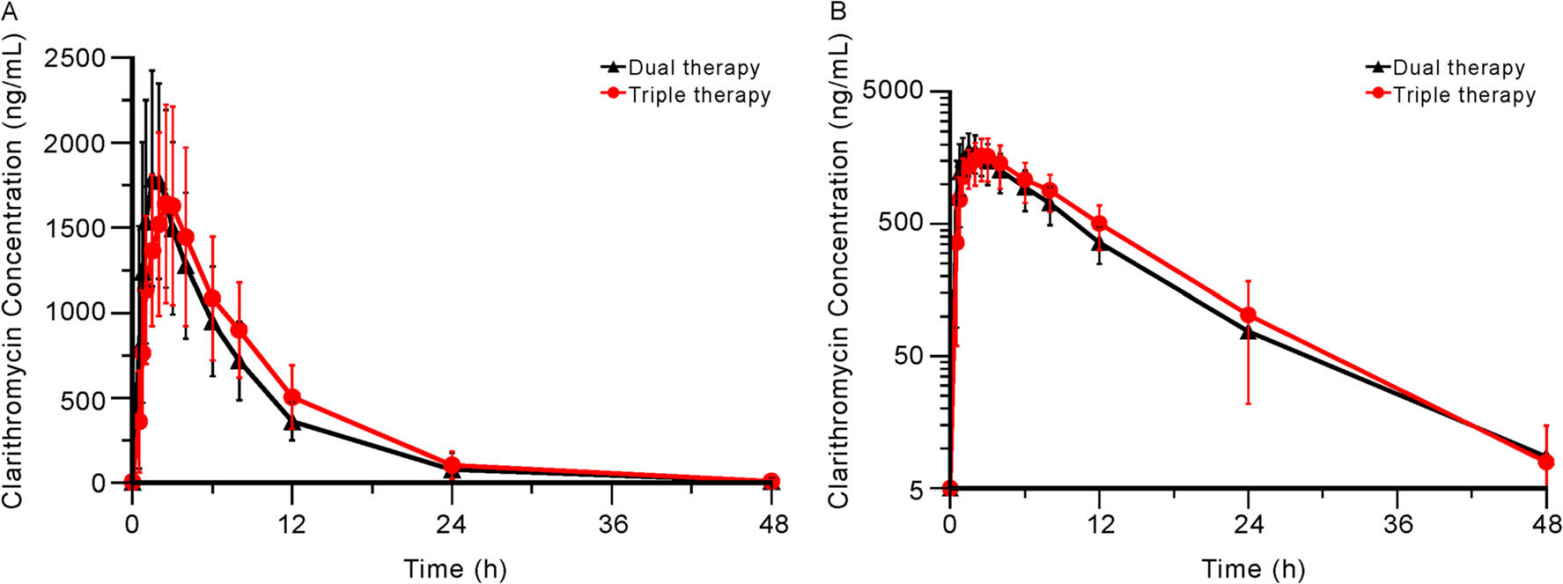
Mean (SD) plasma concentration-time profiles of clarithromycin when administered as dual therapy and as part of triple therapy. **a** Linear scale; **b** semi-logarithmic scale. Abbreviations: dual therapy, administration of clarithromycin 500 mg and amoxicillin 1000 mg in period 1 of treatment part 2; triple therapy, administration of ilaprazole 10 mg, clarithromycin 500 mg, and amoxicillin 1000 mg in period 2 of both treatment parts 1 and 2

**Fig. 4:**
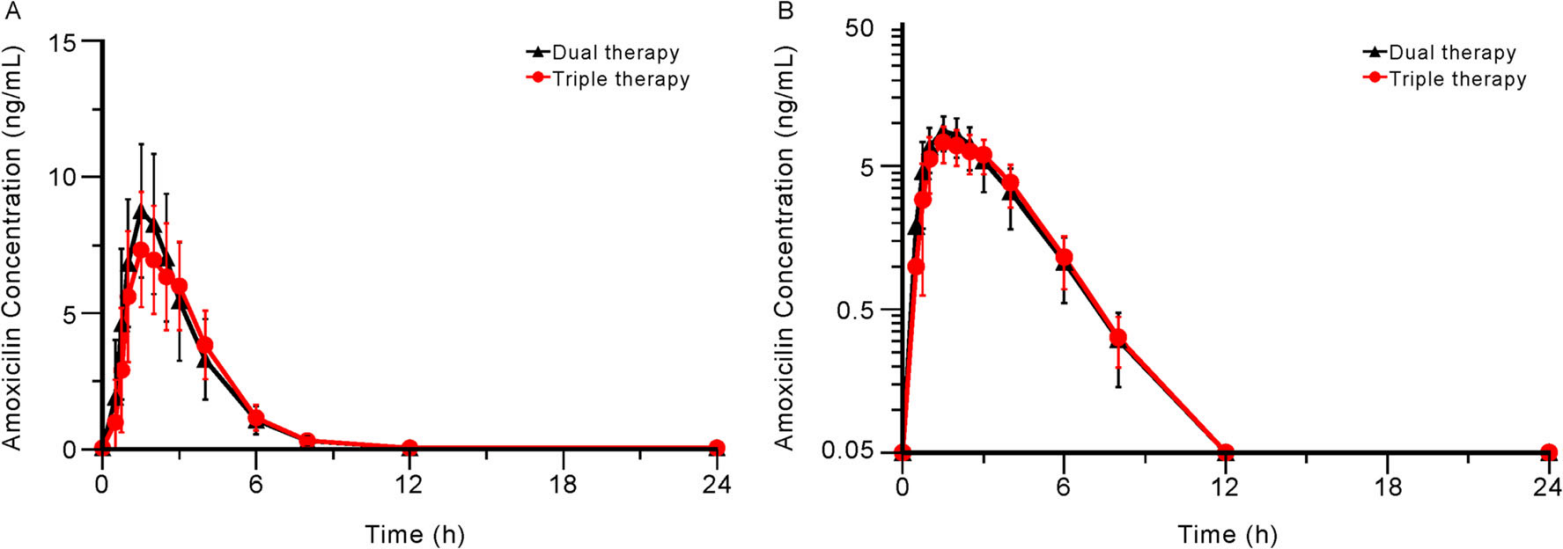
Mean (SD) plasma concentration-time profiles of amoxicillin when administered as dual therapy and as part of triple therapy. **a** Linear scale; **b** semi-logarithmic scale. Abbreviations: dual therapy, administration of clarithromycin 500 mg and amoxicillin 1000 mg in period 1 of treatment part 2; triple therapy, administration of ilaprazole 10 mg, clarithromycin 500 mg, and amoxicillin 1000 mg in period 2 of both treatment parts 1 and 2. Notes: Plasma amoxicillin concentrations at 48 h after study drug administration were not detected

The mean plasma concentration-time profiles of ilaprazole, clarithromycin, and amoxicillin are shown in Figs. 2, 3, and 4. The PK parameters of each study drug and the point estimate with the 90% CI of the GMR of triple therapy to monotherapy or dual therapy are summarised in Table 1.

**Table 1:** PK parameters of ilaprazole, clarithromycin, and amoxicillin following single (or dual) and triple therapy

	Arithmetic mean (SD)		Geometric least square mean		GMR^a^ (90% CI)
Parameters	Monotherapy (or dual therapy)	Triple therapy	Monotherapy (or dual therapy)	Triple therapy	Triple therapy /(monotherapy or dual therapy)
Ilaprazole (*N* = 13)					
T_max_, h	3.5 (2.5–6.0)	3.0 (2.5–8.0)			
C_max_, ng/mL	479 (172)	446 (168)	447	414	0.93 (0.70–1.22)
AUC_last_, h·μg/mL	3301 (1058)	3538 (1198)	3137	3350	1.07 (0.85–1.35)
AUC_inf_, h·μg/mL	3393 (1088)	3681 (1242)	3225	3487	1.08 (0.86–1.36)
t_1/2_, h	4.16 (0.54)	4.66 (0.81)			
CL/F, L/h	3.28 (1.23)	3.04 (1.11)			
Vd/F, L	19.5 (7.1)	20.4 (9.0)			
Clarithromycin (*N* = 15)					
T_max_, h	2.0 (1.0–2.5)	2.5 (1.5–3.0)			
C_max_, μg/mL	1.87 (0.60)	1.72 (0.60)	1.77	1.59	0.90 (0.70–1.15)
AUC_last_, h·μg/mL	14.6 (4.3)	16.5 (6.4)	14.0	15.3	1.09 (0.87–1.37)
AUC_inf_, h·μg/mL	15.0 (4.4)	16.9 (6.4)	14.4	15.7	1.09 (0.87–1.37)
t_1/2_, h	4.84 (2.55)	4.83 (1.10)			
CL/F, L/h	36.6 (12.7)	34.4 (15.1)			
Vd/F, L	247 (118)	230 (89)			
Amoxicillin (*N* = 15)					
T_max_, h	1.5 (0.8–2.5)	2.0 (1.0–3.0)			
C_max_, μg/mL	9.37 (2.09)	8.14 (2.04)	9.14	7.91	0.86 (0.74–1.01)
AUC_last_, h·μg/mL	27.9 (8.4)	26.7 (5.4)	26.7	26.2	0.98 (0.83–1.16)
AUC_inf_, h·μg/mL	28.5 (8.6)	27.4 (5.8)	27.2	26.9	0.99 (0.83–1.17)
t_1/2_, h	1.18 (0.12)	1.96 (3.26)			
CL/F, L/h	38.6 (13.2)	38.0 (7.9)			
Vd/F, L	64.7 (20.1)	95.1 (126)			

Notes: Data are summarised as arithmetic mean ± standard deviation values except those for T_max_, for which median [min–max] values are presented

Abbreviations: *T*_*max*_, time to C_max_; *C*_*max*_, the maximum concentration of drug; *AUC*_*last*_, area under the plasma concentration-time curve from the time of dosing to the last measurable concentration; *AUC*_*inf*_, area under the plasma concentration-time curve from dosing time extrapolated to infinity; *t*_*1/2*_, elimination half-life; *CL/F*, apparent clearance; *Vd/F*, apparent volume of distribution; *monotherapy*, administration of ilaprazole 10 mg in period 1 of part 1; *dual therapy*, administration of clarithromycin 500 mg and amoxicillin 1000 mg in period 1 of part 2; *triple therapy*, administration of ilaprazole 10 mg, clarithromycin 500 mg, and amoxicillin 1000 mg in period 2 of both part 1 and part 2; *GMR*, geometric mean ratio; *CI*, confidence interval

^a^Geometric mean ratio of triple therapy to monotherapy or dual therapy

The mean C_max_ values of ilaprazole administered as part of the triple therapy compared to the monotherapy were 446 and 479 ng/mL, and the mean T_max_ values were 3.0 and 3.5 h after dosing, respectively. The corresponding mean AUC_last_ values was 3538 and 3301 ng·h/mL, and the mean AUC_inf_ values were 3681 and 3393 ng·h/mL, respectively. Although the C_max_ of ilaprazole slightly decreased and the AUC_last_ slightly increased with the triple therapy, those changes were not statistically significant.

The mean C_max_ of clarithromycin was 1.72 and 1.87 μg/mL with the triple and dual therapies, respectively. The mean T_max_, AUC_last_, and AUC_inf_ values of the triple and dual therapies were 2.5 and 2.0 h after dosing, 16.5 and 14.6 μg·h/mL, and 16.9 and 15.0 μg·h/mL, respectively. Similar to the changes in PK data of ilaprazole, the C_max_ of clarithromycin slightly decreased, and the AUC_last_ slightly increased; however, the changes were not statistically significant.

For amoxicillin, the mean C_max_ values of amoxicillin were 8.14 and 9.37 μg/mL with the triple and dual therapies. Furthermore, the corresponding mean T_max_, AUC_last_, AUC_inf_ values for the triple and dual therapy were 2.0 and 1.5 h after dosing, 26.7 and 27.9 μg·h/mL, and 27.4 and 28.5 μg·h/mL, respectively. The C_max_ and AUC_last_ of amoxicillin slightly decreased with the triple therapy. However, the changes were also not statistically significant.

All three drugs were determined not to be significantly affected by the triple coadministration, because the changes in PK data of each drug were clinically and statistically insignificant.

### Safety assessment

A total of 35 AEs were reported by 20 subjects (62.5%), and they are summarised in Table 2. Nine of them (28.1%) were considered to be related to the study drugs, and there was no drug-related AE when subjects received ilaprazole alone. The incidence of drug-related AEs increased with the administration of the triple therapy in each part, but it was not clinically significant since the symptoms were mild and transient. There were no serious AEs, and all subjects recovered without complications. There were no clinically significant differences in vital signs, physical examinations, laboratory tests, and other observations related to safety in both parts. The AEs reported after the administration of the investigational products were generally consistent with the known safety profiles of ilaprazole, clarithromycin, and amoxicillin.

**Table 2:** Summary of adverse events by each part

	Treatment				
	Part I		Part II		Total (*N* = 32)
	Monotherapy (*N* = 16)	Triple therapy (*N* = 13)	Dual therapy (*N* = 16)	Triple therapy (*N* = 15)	
Subjects with any adverse event	5 (31.3)	8 (61.5)	5 (31.3)	7 (46.7)	20^a^ (62.5)
Subjects with adverse drug reactions	–	6 (46.2)	2 (12.5)	3 (20.0)	9^b^ (28.1)
System organ class^a^/preferred term^a^					
Gastrointestinal disorders	–	5 (38.5)	1 (6.3)	1 (6.7)	7 (21.9)
Abdominal discomfort	–	1 (7.7)	–	1 (6.7)	2 (6.3)
Dyspepsia	–	1 (7.7)	–	–	1 (3.1)
Nausea	–	1 (7.7)	1 (6.3)	–	2 (6.3)
Abdominal pain	–	1 (7.7)	–	–	1 (3.1)
Diarrhoea	–	1 (7.7)	–	–	1 (3.1)
Blood and lymphatic system disorders	–	1 (7.7)	–	–	1 (3.1)
Leukopenia		1 (7.7)	–	–	1 (3.1)
Skin and subcutaneous tissue disorders	–	–	–	3 (20.0)	3 (9.4)
Pruritus	–	–	–	2 (13.3)	2 (6.3)
Rash	–	–	–	1 (6.7)	1 (3.1)
Metabolism and nutrition disorders	–	–	1 (6.3)	1 (6.7)	1^c^ (3.1)
Hypertriglyceridemia	–	–	1 (6.3)	1 (6.7)	1^c^ (3.1)

Notes: Data are presented as the number (%) of subjects with adverse events or adverse drug reactions

Abbreviations: *monotherapy*, administration of ilaprazole 10 mg in period 1 of part 1; *dual therapy*, administration of clarithromycin 500 mg and amoxicillin 1000 mg in period 1 of part 2; *triple therapy*, administration of ilaprazole 10 mg, clarithromycin 500 mg, and amoxicillin 1000 mg in period 2 of both part 1 and part 2

^a^A total of 5 subjects showed adverse events in both treatment groups in each part

^b^Two subjects developed adverse drug reactions in both treatment groups in the relevant part

^c^One subject developed adverse drug reaction in both treatment groups in the relevant part

## Discussion

This study showed that there were no significant PK drug interactions between clarithromycin or amoxicillin and ilaprazole. Based on the results from in vitro studies that ilaprazole is mainly metabolised by CYP3A4 among CYP-isofoms, our result demonstrating that clarithromycin showed no significant drug interaction with ilaprazole poses a question. However, it does not negate that clarithromycin influences the metabolism of ilaprazole. In our result, the exposure and elimination of ilaprazole were slightly affected by the triple therapy. The AUC_last_ slightly increased, the half-life was slightly prolonged, and the clearance (CL/F) was slightly reduced, although those findings were not of a statistical significance. It suggests that the metabolism of ilaprazole might be influenced by clarithromycin, but within a small extent. The influence of clarithromycin on the exposure of ilaprazole could be limited if another metabolic pathway (e.g. non-enzymatic pathway) plays a considerable part in the metabolism of ilaprazole. Although we did not measure the concentration of metabolites in this study, previous studies revealed that ilaprazole sulfone and thiol ether were detected in comparable amounts when ilaprazole was administered to human [20, 21]. This implies that non-enzymatic pathway might not be minor. Taken together, these results led us to speculate that clarithromycin might affect the metabolism of ilaprazole, but its influence was limited, because ilaprazole could be metabolised by non-enzymatic pathway.

Regarding drug interaction in the absorption of ilaprazole, a previous study by Cao et al. reported that the C_max_ and the AUC of ilaprazole and ilaprazole sulfone significantly decreased following the triple therapy, while ilaprazole thiol ether, another metabolite of ilaprazole, did not change [20]. Cao et al. speculated that the decrease in absorption of ilaprazole was the mechanism of their result, because the half-life and the clearance of ilaprazole were not affected. However, we could not identify a significant decrease in the absorption of ilaprazole in our study, although its C_max_ slightly decreased. We suggest that follow-up studies are needed to validate the influence of clarithromycin or amoxicillin on the absorption of ilaprazole.

Proton pump inhibitors may also have an inhibitory effect on CYP3A4 activity and increase the exposure of drugs metabolised mainly by CYP3A4 [11]. However, this effect varies among PPIs. It has been revealed that omeprazole and lansoprazole increase the exposure of clarithromycin when coadministered [8, 9]. Pantoprazole was shown to be a competitive inhibitor of CYP3A4 via in vitro study, but the metabolic pathway has not yet been demonstrated in human studies [11]. Rabeprazole did not show any inhibitory effect on CYP isoforms, but the non-enzymatically formed metabolite of rabeprazole, rabeprazole thioether, was revealed to have an inhibitory effect on CYP2C9, CYP2C19, CYP2D6, and CYP3A4 [28]. However, the plasma concentration of clarithromycin was largely unchanged by combination therapy with esomeprazole [10]. As in the case of esomeprazole, the exposure of clarithromycin was not significantly affected by ilaprazole in our study.

In addition to the anti-acid secretory effect, PPIs are considered to enhance the anti-*H. pylori* activities of concomitantly administered amoxicillin and clarithromycin in *H. pylori* eradication therapy. It has been demonstrated that PPIs enhance the stability of acid-labile antibiotics (e.g. penicillins) by increasing the gastric pH [12]. Based on this finding, it has been speculated that PPIs may increase the bioavailability of amoxicillin in triple therapy [11]. Despite this prediction, there are no reports that demonstrate any significant changes in the exposure of amoxicillin in the drug interaction studies between amoxicillin and PPIs [9, 10, 20, 29, 30]. Our result also showed no clinically significant changes in the exposure of amoxicillin except a slight decrease in the C_max_ (GMR, 0.86; 90% CI, 0.74–1.01). It would be difficult to demonstrate further increase in amoxicillin exposure by increasing pH, considering already high bioavailability of amoxicillin (89–98%) [31] and the single dosing of amoxicillin in our study.

Although someone might speculate that the PK interactions between PPIs and clarithromycin resulting in increased exposures of both drugs may contribute to higher eradication rates [20], drug interactions causing excessive exposure may be detrimental to defining the appropriate dosage regimen and may result in undesirable increases in adverse events. In this respect, our results support that ilaprazole, the exposure of which is only minimally influenced by coadministration of clarithromycin and amoxicillin, may have advantages over other PPIs to be used in triple therapy regimen.

Taken together, the results of our study indicate that there were no clinically significant PK drug interactions between clarithromycin, amoxicillin, and ilaprazole. Thus, the standard triple therapy with ilaprazole could be a desirable treatment option for *H. pylori* eradication. The limitation of our study is that the subjects were limited to healthy male Korean population. Because there are relevant ethnicity-related differences in PPI metabolism [32], further studies are required to evaluate whether there is an ethnicity- or sex-related difference in the drug interactions of ilaprazole.

## Conclusions

The coadministration of ilaprazole, clarithromycin, and amoxicillin was tolerable and did not cause a significant PK drug interaction. Thus, a triple therapy regimen comprising ilaprazole, clarithromycin, and amoxicillin may be an option for the eradication of *H. pylori*.

## Electronic supplementary material

Supplementary Table 1 Demographics of study participants in each part (DOCX 17 kb)

## References

[1] Malfertheiner P, Chan FK, McColl KE (2009). Peptic ulcer disease. Lancet.

[2] Lee DH (2002). Current status and treatment of Helicobacter pylori infection in Korea. Korean J Gastroenterol.

[3] Rauws EA, Tytgat GN (1990). Cure of duodenal ulcer associated with eradication of *Helicobacter pylori*. Lancet.

[4] Malfertheiner P, Leodolter A, Peitz U (2000). Cure of *Helicobacter pylori*-associated ulcer disease through eradication. Baillieres Best Pract Res Clin Gastroenterol.

[5] Kim N, Kim JJ, Choe YH, Kim HS, Kim JI, Chung IS (2009). Diagnosis and treatment guidelines for *Helicobacter pylori* infection in Korea. Korean J Gastroenterol.

[6] Sasaki M, Ogasawara N, Utsumi K, Kawamura N, Kamiya T, Kataoka H, Tanida S, Mizoshita T, Kasugai K, Joh T (2010). Changes in 12-year first-line eradication rate of *Helicobacter pylori* based on triple therapy with proton pump inhibitor, amoxicillin and clarithromycin. J Clin Biochem Nutr.

[7] Jung YS, Park CH, Park JH, Nam E, Lee HL (2017) Efficacy of *Helicobacter pylori* eradication therapies in Korea: a systematic review and network meta-analysis. Helicobacter 22 10.1111/hel.12389 28425141

[8] Calabresi L, Pazzucconi F, Ferrara S, Di Paolo A, Tacca MD, Sirtori C (2004). Pharmacokinetic interactions between omeprazole/pantoprazole and clarithromycin in health volunteers. Pharmacol Res.

[9] Mainz D, Borner K, Koeppe P, Kotwas J, Lode H (2002). Pharmacokinetics of lansoprazole, amoxicillin and clarithromycin after simultaneous and single administration. J Antimicrob Chemother.

[10] Hassan-Alin M, Andersson T, Niazi M, Liljeblad M, Persson BA, Rohss K (2006). Studies on drug interactions between esomeprazole, amoxicillin and clarithromycin in healthy subjects. Int J Clin Pharmacol Ther.

[11] Ogawa R, Echizen H (2010). Drug-drug interaction profiles of proton pump inhibitors. Clin Pharmacokinet.

[12] Goddard AF, Jessa MJ, Barrett DA, Shaw PN, Idstrom JP, Cederberg C, Spiller RC (1996). Effect of omeprazole on the distribution of metronidazole, amoxicillin, and clarithromycin in human gastric juice. Gastroenterology.

[13] de Bortoli N, Martinucci I, Giacchino M, Blandizzi C, Marchi S, Savarino V, Savarino E (2013). The pharmacokinetics of ilaprazole for gastro-esophageal reflux treatment. Expert Opin Drug Metab Toxicol.

[14] Du YQ, Guo WY, Zou DW (2012). Acid inhibition effect of ilaprazole on Helicobacter pylori-negative healthy volunteers: an open randomized cross-over study. J Dig Dis.

[15] Periclou AP, Goldwater R, Lee SM, Park DW, Kim DY, Cho KD, Boileau F, Jung WT (2000). A comparative pharmacodynamic study of IY-81149 versus omeprazole in patients with gastroesophageal reflux disease. Clin Pharmacol Ther.

[16] Vallve M, Vergara M, Gisbert JP, Calvet X (2002). Single vs. double dose of a proton pump inhibitor in triple therapy for *Helicobacter pylori* eradication: a meta-analysis. Aliment Pharmacol Ther.

[17] Li Y, Zhang W, Guo D, Zhou G, Zhou H, Xiao Z (2008). Pharmacokinetics of the new proton pump inhibitor ilaprazole in Chinese healthy subjects in relation to CYP3A5 and CYP2C19 genotypes. Clin Chim Acta.

[18] Myung SW, Min HK, Jin C, Kim M, Lee SM, Chung GJ, Park SJ, Kim DY, Cho HW (1999). Identification of IY81149 and its metabolites in the rat plasma using the on-line HPLC/ESI mass spectrometry. Arch Pharm Res.

[19] Seo KA, Lee SJ, Kim KB, Bae SK, Liu KH, Kim DH, Shin JG (2012). Ilaprazole, a new proton pump inhibitor, is primarily metabolized to ilaprazole sulfone by CYP3A4 and 3A5. Xenobiotica.

[20] Cao S, Zhou G, Ou-Yang DS (2012). Pharmacokinetic interactions between ilaprazole and clarithromycin following ilaprazole, clarithromycin and amoxicillin triple therapy. Acta Pharmacol Sin.

[21] Zhou G, Tan ZR, Zhang W, Ou-Yang DS, Chen Y, Guo D, Liu YZ, Fan L, Deng HW (2009). An improved LC-MS/MS method for quantitative determination of ilaprazole and its metabolites in human plasma and its application to a pharmacokinetic study. Acta Pharmacol Sin.

[22] Ho KY, Kuan A, Zano F (2009). Randomized, parallel, double-blind comparison of the ulcer-healing effects of ilaprazole and omeprazole in the treatment of gastric and duodenal ulcers. J Gastroenterol.

[23] Song J, Guo B, Yao L, Tang J (2010). S1046 the clinical study of Ilaprazole on duodenal ulcer, a randomize study compared with esomeprazole. Gastroenterology.

[24] U.S. Food and Drug Administration. Amoxil capsules, Prescribing information. https://www.accessdata.fda.gov/drugsatfda_docs/label/2008/050542s24,050754s11,050760s10,050761s10lbl.pdf. Accessed Nov 24 2017

[25] U.S. Food and Drug Administration. Biaxin tablet, Prescribing Information. https://www.accessdata.fda.gov/drugsatfda_docs/label/2009/050662s042,050698s024,050775s013lbl.pdf

[26] Wang L, Zhou L, Hu H, Lin S, Xia J (2012). Ilaprazole for the treatment of duodenal ulcer: a randomized, double-blind and controlled phase III trial. Curr Med Res Opin.

[27] Xue Y, Qin X, Zhou L, Lin S, Wang L, Hu H, Xia J (2016). A randomized, double-blind, active-controlled, multi-center study of Ilaprazole in the treatment of reflux esophagitis. Clin Drug Investig.

[28] Li XQ, Andersson TB, Ahlstrom M, Weidolf L (2004). Comparison of inhibitory effects of the proton pump-inhibiting drugs omeprazole, esomeprazole, lansoprazole, pantoprazole, and rabeprazole on human cytochrome P450 activities. Drug Metab Dispos.

[29] Furuta T, Shirai N, Takashima M, Xiao F, Hanai H, Sugimura H, Ohashi K, Ishizaki T, Kaneko E (2001). Effect of genotypic differences in CYP2C19 on cure rates for *Helicobacter pylori* infection by triple therapy with a proton pump inhibitor, amoxicillin, and clarithromycin. Clin Pharmacol Ther.

[30] Gustavson LE, Kaiser JF, Edmonds AL, Locke CS, DeBartolo ML, Schneck DW (1995). Effect of omeprazole on concentrations of clarithromycin in plasma and gastric tissue at steady state. Antimicrob Agents Chemother.

[31] Sanchez Navarro A (2005). New formulations of amoxicillin/clavulanic acid: a pharmacokinetic and pharmacodynamic review. Clin Pharmacokinet.

[32] Chong E, Ensom MH (2003). Pharmacogenetics of the proton pump inhibitors: a systematic review. Pharmacotherapy.

