# Supplementary material for: Pharmacokinetic drug interaction and safety after coadministration of clarithromycin, amoxicillin, and ilaprazole: a randomised, open-label, one-way crossover, two parallel sequences study
Source: Eur J Clin Pharmacol. 2018 May 30;74(9):1149–57. doi: 10.1007/s00228-018-2489-2 (PMC6096703; doi:10.1007/s00228-018-2489-2)
Supplement: Supplementary file 1 — Demographics of study participants in each part (DOCX 17 kb) [file 228_2018_2489_MOESM1_ESM.docx]

Supplementary Table 1. Demographics of study participants in each part

| **Variables** | **Part 1**  **(*n* = 16)** | **Part 2**  **(*n* = 16)** | **Total**  **(*N* = 32)** | ***P* value** |
| --- | --- | --- | --- | --- |
| Age (years) | 28.4 ± 5.8 | 28.1 ± 5.8 | 28.3 ± 5.7 | 0.90 |
| Height (cm) | 175.0 ± 5.3 | 176.6 ± 5.2 | 175.8 ± 5.2 | 0.39 |
| Weight (kg) | 68.1 ± 7.9 | 68.4 ± 6.9 | 68.3 ± 7.3 | 0.92 |
| BMI (kg/m^2^) | 22.1 ± 1.8 | 21.9 ± 2.0 | 22.0 ± 1.9 | 0.69 |

Notes: Data are summarized as arithmetic mean ± standard deviation. *P* values were derived using the Student’s *t* test. The normal distribution of the data was comprehensively evaluated using the Shapiro-Wilk test and q-q plot. Subjects assigned to part 1 received ilaprazole 10 mg on day 1 of period 1 and clarithromycin 500 mg and amoxicillin 1000 mg from day 1 to 6 of period 2 and ilaprazole 10 mg was co-administered on day 5 of period 2. Subjects assigned to part 2 received both clarithromycin 500 mg and amoxicillin 1000 mg on day 1 of period 1 and ilaprazole 10 mg from day 1 to 6 of period 2 and clarithromycin 500 mg and amoxicillin 1000 mg was co-administered on day 5 of period 2.

Abbreviation: BMI, body mass index
